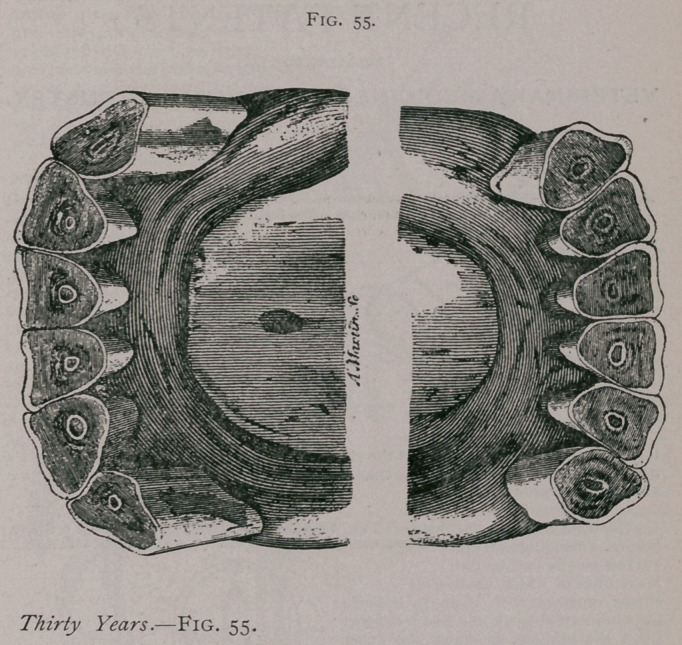# Age of the Horse, Ox, Dog and Other Domestic Animals

**Published:** 1891-02

**Authors:** R. S. Huidekoper

**Affiliations:** Veterinarian


### AGE OF THE HORSE, OX, DOG, AND OTHER DOMESTICATED ANIMALS.

BY R. S. HUIDEKOPER, M. D., VETERINARIAN.

[*Continued from page 29.*]

FIG. 53.

Nineteen years.

FIG. 53.

*Nineteen Years.*—FIG 53.

From in front the superior corner teeth point distinctly toward the median line, the intermediate teeth commence to incline in the same direction, showing a marked triangular space between them at the line of the gums. In Profile the ogive formed by the apposition of the jaws is more closed. The notch still remains in the superior corner teeth but is less marked in this age from the increased horizontal position of the inferior corner teeth. The tables of the pincers and the intermediate inferior teeth seem to converge at their posterior border on account of their diverging in front; their antero-posterior diameters have become greater. The inferior corners are always triangular in the upper jaw; the pincher teeth are usually leveled although the cup sometimes remain in them for several years more.

FIG. 54.

Twenty-one years.

FIG. 54.

*Twenty-one Years.*—FIG. 54.

The teeth become so horizontal that, looked at in front, the inferior ones are scarcely seen unless the head is well raised. The triangular interspaces at the base of the superior incisors increase in size and the convergence of the free ends becomes more marked. In profile the jaws are seen diminished in size; the inferior corners have become almost horizontal and have worn off the notch on the superior corner.

The wearing surfaces of these teeth become elongated from in front to behind and have lost their triangular shape. The tables of the superior pincers and intermediate teeth are elongated from in front to behind and distinctly triangular; they are generally leveled. The tables of the inferior teeth commence to flatten from side to side and are more distinctly separated.

FIG. 55.

Thirty years.

FIG. 55.

*Thirty Years.*—FIG. 55.

This plate shows the character of extreme old age. In front the superior incisive arch over-laps the inferior, which has become considerably narrower, the convergence of the corner and intermediate teeth becomes more marked. In profile the inferior incisors are almost horizontal especially the corner ones; the jaws are thinned and are wider apart from each other in the region of the bars. The tables of the inferior teeth are flattened from side to side (biangular). The peripheral enamel has almost disappeared from the posterior border of the teeth. In the upper jaw the tables are flattened from side to side and the enamel is nearly worn away. Sometimes in one or in both jaws the teeth have acquired an extreme length and are not leveled; or, at other times they are worn away almost to the roots and level with the gums, and are surrounded by a large deposit of the radical cement which covers the dentine, while the enamel has entirely disappeared.

[TO BE CONTINUED.]

## Figures and Tables

**Fig. 53. f1:**
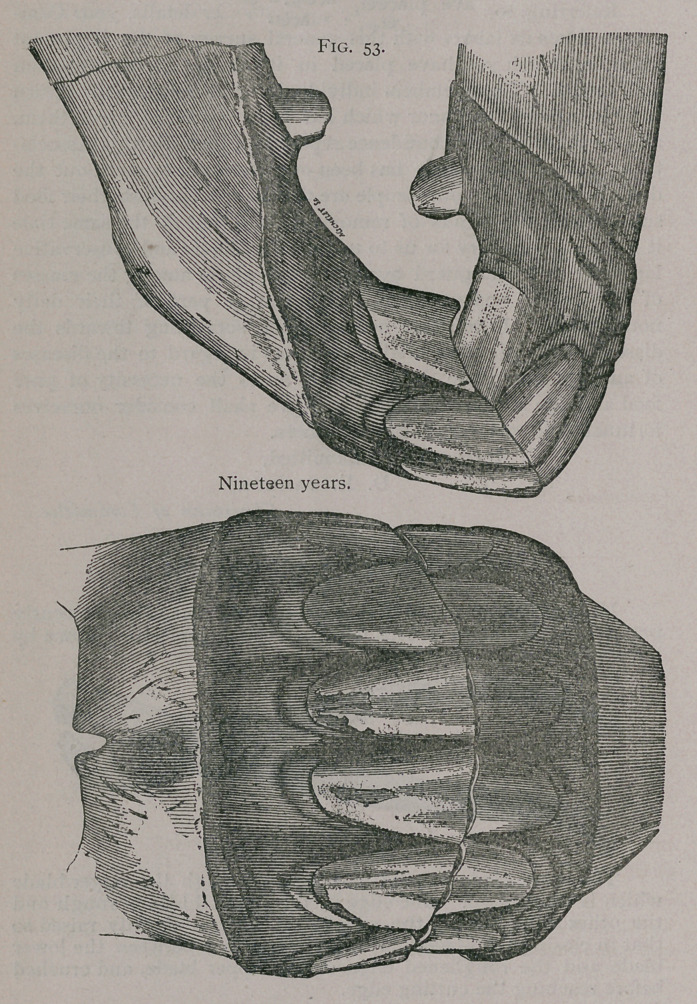


**Fig. 53. f2:**
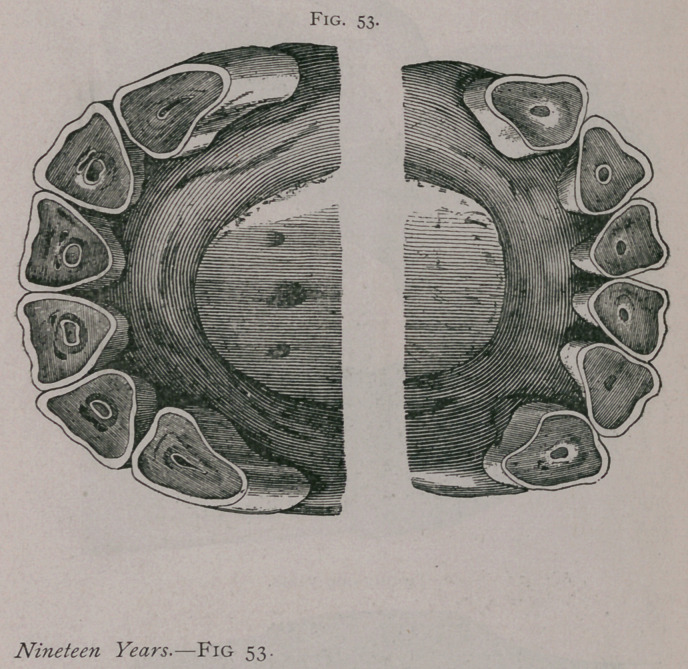


**Fig. 54. f3:**
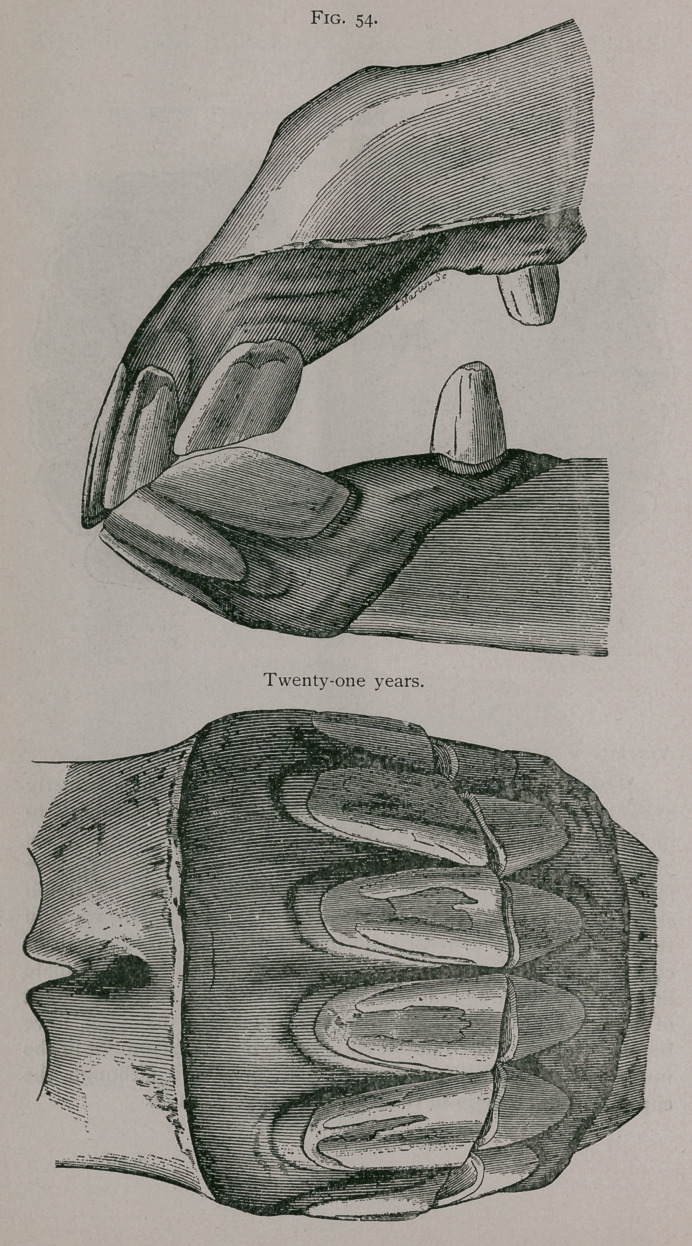


**Fig. 54. f4:**
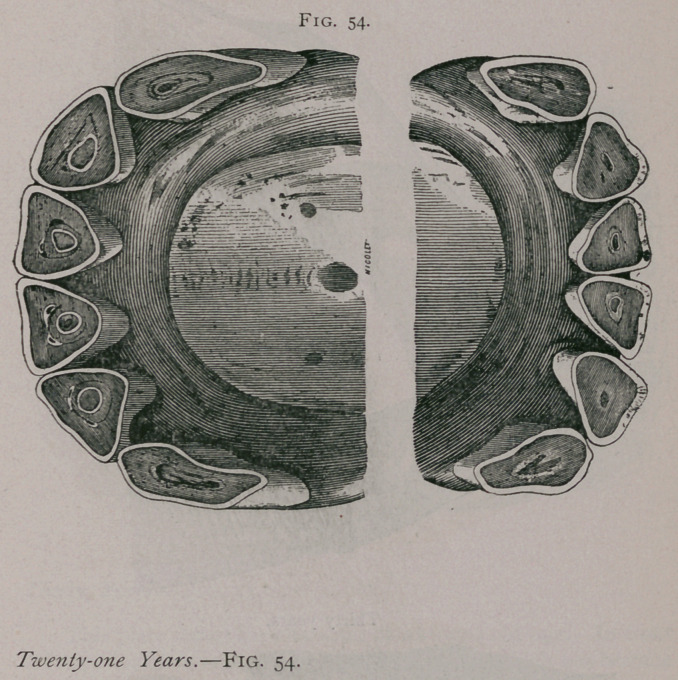


**Fig. 55. f5:**
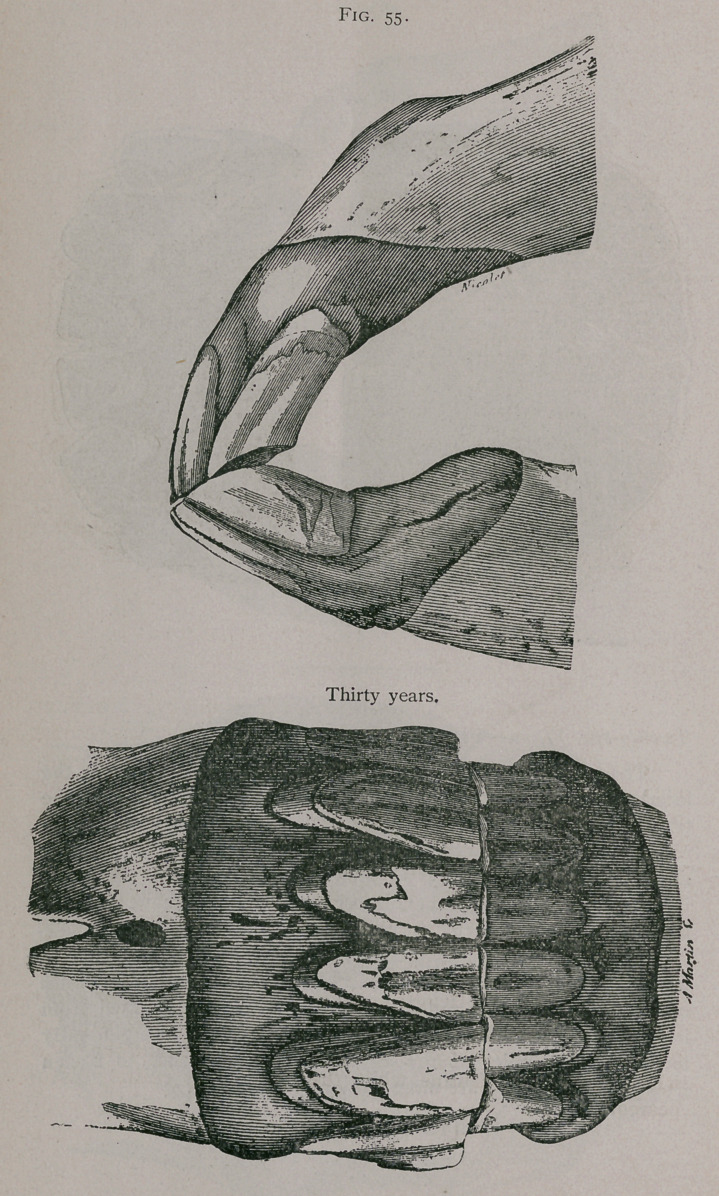


**Fig. 55. f6:**